# Perinatal/maternal-fetal-infant dermatologic manifestations of SARS-CoV-2. An Overview and Implications for diagnosis, treatment, and prognosis

**DOI:** 10.3389/fped.2022.1071839

**Published:** 2022-12-02

**Authors:** Elaine M. Young

**Affiliations:** ^1^ Adult and Pediatric Dermatology, Private Practice, Huntington, WV, United States; ^2^ Department of Internal Medicine, Joan C. Edwards School of Medicine, Huntington, WV, United States

**Keywords:** SARS-CoV-2, pregnant women, neonates, infants, dermatologic patterns, skin

## Abstract

Early identification of the dermatologic manifestations of SARS-CoV-2 in perinatal and maternal-fetal-infant populations is essential for early intervention in the diagnosis, treatment, and prevention of short and long term sequelae. Although cutaneous signs of SARS-CoV-2 are less common in pregnant women, neonates, and infants, the recognition of related skin lesions with regard to timing, location, duration, and pattern can lead to determining disease severity. While many pediatric patients may be asymptomatic with negative SARS-CoV-2 testing, skin lesions may be the only clue of infection. SARS-CoV-2 infection in pregnancy can lead to severe life threatening illness and by understanding the cutaneous manifestations associated with SARS-CoV-2 infection, early diagnosis can be made with improved maternal-fetal outcomes. A wide array of dermatologic presentations associated with SARS-CoV-2 are reported in the literature. This review explores the expanding reports in the literature of the dermatologic presentations of skin lesions related to SARS-CoV-2 specifically in perinatal and maternal-fetal-infant health and the implications for management. The collaboration of the specialties of dermatology, pediatrics, obstetrics/gynecology, and infectious disease in the approach to SARS-CoV-2 disease can lead to a better understanding of the scope and presentation of this disease.

## Introduction

Since the first report and isolation of SARS-CoV-2 infection in December 2019 in Wuhan, China, more than 600 million people have been infected globally causing over 6.4 million deaths (1). The ability to predict SARS-CoV-2 disease course and prevent transmission remains challenging but identifying dermatologic manifestations may have diagnostic and prognostic implications. Early reports of adverse effects associated with pregnancy were scarce but recent comparison studies present evidence that pregnant women with SARS-CoV2 have an increased susceptibility to hospitalization and severe illness (2, 3). The incidence of neonatal and infant SARS-CoV-2 infection is less common than adults but when infected have the potential for serious complications (3–5).

Dermatologic manifestations of SARS-CoV-2 were first reported in March 2020 by Recalcati (6) with the description of infected patients presenting with an erythematous vesicular and urticarial eruption. Subsequently, multiple varying presentations in infected patients were eventually categorized into distinct patterns. Certain types of skin patterns are associated with more severe SARS-CoV-2 infections and can help establish the timeline of the disease process. Skin manifestations of SARS-CoV2 must be differentiated from diseases that normally be seen or exacerbated in pregnant women, neonates, and infants. A team approach of dermatologists, obstetricians, neonatologists, pediatricians and infectious disease specialists is ideal to optimize patient care.

## Immunology

There is a complex interplay of physiologic immunological responses in healthy pregnant women, neonates, and infants that may affect SARS-CoV-2 susceptibility and skin disease presentation (7). Natural immunological shifts in pregnancy to protect the fetus result in down regulation of cell mediated immunity and upregulation of humoral immunity responses. The results are decreased T helper 1 cell (Th1) cytokine production (interleukin-12 (IL-12), interferon-gamma (IFN γ) and increased T helper 2 cell cytokines (IL-4, IL-10) (7, 8). Cytokines are needed for cell signaling and development of healthy neonates and infants especially with respect to adaptive immune responses. Innate decreased expression of IFN γ and tumor necrosis factor-alpha (TNF-α) in neonates and infants is postulated to be associated with increased susceptibility to infection (8–11). Physiologic cytokine alterations may lead to exacerbation of skin diseases in pregnancy (10).

Serious SARS-CoV-2 complications are attributed to a viral stimulated hyperinflammatory state leading to immunological responses and an exaggerated release of cytokines (“Cytokine Storm”) (12). Among the main inflammatory mediators associated in this process are IFN γ, IL-6, and TNF-α, prime mediators involved in the physiological immune shifts in pregnant women, neonates and infants and in the pathogenesis of certain SARS-CoV-2 skin manifestations (13, 14). Tanacan et al. (13) reported in a study of 90 SARS-CoV-2 infected pregnant women that severity of illness correlated with elevation of IFN γ, IL-6 and D-Dimer and lower levels of IL-2, IL-10, and IL-17.

The combination of immunologic responses in pregnant women, neonates and infants with SARS-CoV-2 and skin disease results in a challenging complicated clinical picture created by the interactions of cytokines and pathophysiologic mechanisms (15).

## Dermatologic patterns

Several main dermatological patterns associated with SARS-CoV-2 have been categorized and should be recognized in pregnant women, neonates and infants (16–20). Although patients with SARS-CoV-2 can present with polymorphic skin lesions, six common patterns have been described in the literature as (1) maculopapular (2) urticarial (3) vesicular (4) chilblain-like (5) livedo and (6) purpuric-vasculitic patterns (19, 20). The first 3 groups comprise lesions that are inflammatory and exanthematous and the latter 3 categories including cutaneous vasculitic disorders and vasculopathies (16, 17). These patterns have been noted in other skin diseases in pregnancy, neonatology and infancy recognizing the importance of a keen differential dermatologic diagnosis. Table 1 summarizes SARS CoV-2 dermatologic patterns and relevant clinical aspects to pregnant women, neonates, and infants.

**Table 1:** Summarizes SARS CoV-2 associated dermatologic patterns and relevant clinical aspects in pregnant women, neonates, and infants.

SARS CoV-2 associated dermatologic patterns: relevance in pregnant women, neonates and infants				
Patterns	Features	Timing	Differential DX	Key points
Maculopapular	Most common Age variable Blanching erythematous macules/papules; +/– pruritus Trunk- diffuse spread	Mid-course/symptomatic (Early/Late onset reported)	Drug, Viral (Parvo B-19, Measles, Rubella, HHV6, Enterovirus, Adenovirus) Streptococcal, Syphilis Pregnancy: PEP, AEP Neonate: ETN Neonate/Infant: Miliaria, Atopic Dermatitis	Favorable prognosis Cases reports: Preterm labor, Premature rupture of membranes, Transplacental viral transmission;? Trigger - antiviral therapy
Urticarial	Age/Prevalence variable Female tendency Transient wheals;++pruritus Trunk to acral/face spread	Prodromal/asymptomatic (Symptomatic pts reported with mod/severe disease)	Drug, Viral (CMV, RSV, EBV, HSV), Mycoplasma, Parasitic, Food, Allergy, Idiopathic Pregnancy: PEP Neonate/Infant: Infections,Food, Drug, Atopy	Can be associated with severe SARS CoV-2 Difficult to differentiate from other infections/drug rx PEP- 3rd trimester, begins abdominal striae, longer duration than SARS COV2 urticaria
Vesicular	Less common Adults-reported in children Vesicles +/− purpura; +/− pruritus Trunk - localized or diffuse	Mid-course/symptomatic (Early/Late onset reported)	VZV, HSV, Enterovirus, Echovirus, Impetigo Scabies, Miliaria, Dermatitis, Drug, Autoimmune Pregnancy: Pemphigoid Gestationis Neonate/Infant: ETN, Acropustulosis of Infancy HyperIgE, Histiocytosis	Specific skin SARS CoV-2 manifestation May be helpful in early diagnosis Can be associated with mod-severe disease Eliminate other viral/bacterial: serious fetal sequelae
Chilblain-like	Prevalence variable Young adults/children Erythematous/violaceous macules papules/nodules Toes/Fingers	Late onset/asymptomatic/ mild disease	Idiopathic or Secondary (Autoimmune-CLE, Antiphospholipid disease), Cryoglobulenemia, Raynaud's disease, Hematologic, Neoplastic	Most familiar skin SARS CoV-2 manifestation Mild disease course -resolves 1-2 weeks Late onset -may be only sign of SARS CoV-2 Evaluate for Hypercoagulable or Autoimmune disease
Livedo	Less common Prevalence- elderly Mottled erythema/violaceous Net-like discoloration/purpura Distal extremities to diffuse spread	Mid-course/symptomatic	Idiopathic, Physiologic, Autoimmune, Hematologic Viral, Bacterial, Drug, Neurologic Pregnancy: SLE, Antiphospholipid Disease, Erythema Ab Igne Neonate/Infant: CMTC, Erythema Ab Igne, HSP	Livedo Reticularis - mild disease course Livedo Racemosa - severe disease course/coagulopathy Livedo Pattern and pregnancy - risk of severe disease Neonate/Infants - Livedo pattern unusual in SARS COV2
Purpuric/vasculitic	Least common Age variable Hemorrhagic macules/papules Palpable purpura Distal extremities to diffuse spread	Mid–course/symptomatic Diffuse = severe infection	Viral (Hepatitis, HIV) Bacterial (Meningococcus) Drug, Hematologic, Autoimmune, Neoplastic Nutritional disorders (Vitamin C deficiency) Pregnancy: SLE, TTP, ANCA-Associated Vasculitis Neonate/Infant: HSP, Kawasaki's Disease, MIS	Severe morbidity/mortality Skin-Prognostic sign Kawaski's like/MIS-C/MIS-N associated = severe disease

AEP, atopic eruption of pregnancy; ANCA, anti-neutrophil cytoplasmic antibody; CLE, cutaneous lupus erythematosus; CMTC, cutis marmorata telangiectatica congenita; CMV, cytomegalovirus; EBV, epstein barr virus; ETN, erythema toxicum neonatorum; HHV6, human herpesvirus 6; HIV, human immunodeficiency virus; HSP, henoch schonlein purpura; HSV, herpes simplex virus; MIS, multisystem inflammatory syndrome; Parvo B19, parvovirus B-19; PEP, polymorphic eruption of pregnancy; RSV, respiratory syncytial virus; SLE, systemic lupus erythematosus; TTP, thrombotic thrombocytopenic purpura; VZV, varicella zoster virus.

### Maculopapular

**Figure 1:**
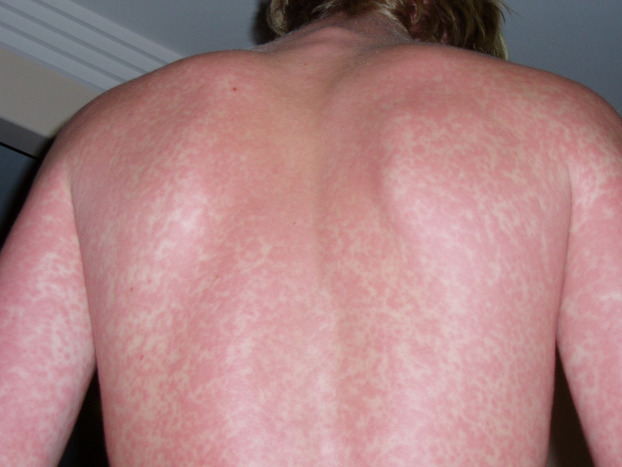
Maculopapular pattern.

Maculopapular exanthems appear to be the most prevalent of all patterns. In a case series of 375 patients with SARS-CoV-2 infection, 47% presented with maculopapular lesions (17). In smaller case studies, the prevalence varied from 5%–70% (19). The exanthem can occur at any age consisting of small red raised and flat lesions which typically appear on the trunk and spread to the extremities but may appear on the face and neck (Figure 1).

The eruption may or may not be associated with pruritus. The lesions are most notably observed during the mid course of infection when the patient is most symptomatic. A few studies have reported a latent onset up to 27 days after diagnosis (21, 22). Variants of this exanthem have been reported as purpuric-like, erythema-multiforme-like, pityriasis-rosea- like, erythema elevatum diutinum-like and perifollicular patterns (23).

The majority of patients with maculopapular lesions tend to have an uneventful course, but there are reports of SARS-CoV-2 infected pregnant women presenting with only maculopapular eruptions devoid of constitutional symptoms with premature rupture of membranes (24). Rare cases of transplacental transmission of SARS CoV-2 have been reported in maternal infections with maculopapular lesions (25–28). Oropez et al. (25) reported a case of a 34 year old pregnant woman with a diamniotic dichorionic twin pregnancy presenting with mild SARS Co-V-2 infection and a maculopapular eruption in the 3rd trimester. Healthy twins delivered by cesarean section revealed one twin was positive for SARS Co-V-2 IgG antibodies while the other twin was serologically negative. Placental pathology was negative for evidence of SARS-Co-V-2. Maculopapular exanthems have been reported in infants with SARS-CoV-2 infection in association with mild symptomatic disease (17). The primary differential diagnosis includes other viral infections or adverse drug eruptions (Table 1). SARS-CoV-2 antiviral therapy can produce drug reactions appearing identical to viral eruptions making identification of the inciting agent challenging (14).

Polymorphic Eruption of Pregnancy (PEP) (Synonym-PUPP Pruritic Urticarial Papules and Plaques of Pregnancy) and Atopic Eruption of Pregnancy (AEP) may present in pregnant women as maculopapular lesions (29). Erythema toxicum neonatorum (ETN), miliaria, and atopic dermatitis could appear with maculopapular lesions in neonates and infants (30, 31). It is imperative to consider SARS CoV-2 infections in the differential of maculopapular eruptions in pregnant women, neonates and infants for proper intervention to prevent complications and transmission.

### Urticarial

**Figure 2:**
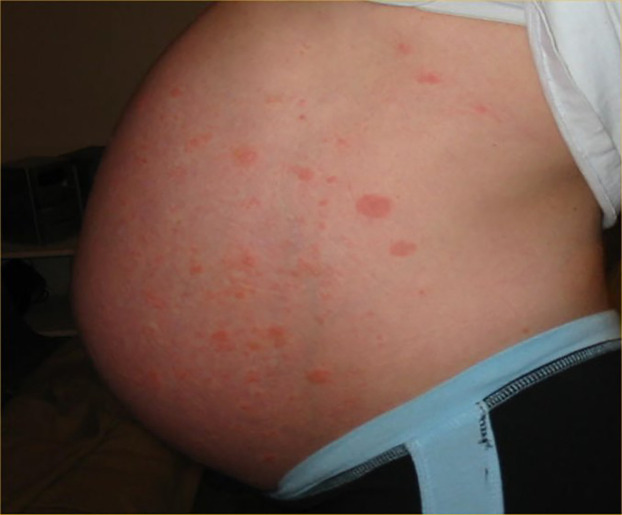
Urticarial pattern.

Urticarial lesions seen in SARS CoV-2 are generally encountered during the prodromal asymptomatic period of disease and may be the first sign of disease (Table 1). The incidence of urticaria in SARS-CoV-2 infection ranges from 16.7%–19% and has a higher prevalence in females (17, 32, 33). Although several studies have reported SARS CoV-2 associated urticaria primarily in adults, several cases have been documented in children (34). The lesions generally last a week and have been associated with moderate to severe complications in some patients (17). Hive-like, blanching thin plaques which are transient and changeable in shape with severe pruritus typically present on the trunk and spread to the extremities possibly affecting the face and acral areas (Figure 2). Angioedema and urticarial vasculitis may also occur (33).

Urticaria secondary to SARS CoV-2 may be difficult to differentiate from other causes such as medications, food, bacterial, parasitic, other viral infections, allergic reactions and idiopathic urticaria (33, 34). Pathophysiologically, SARS-CoV-2 stimulates mast cell degranulation through either direct viral contact or complement activation and cytokine release. It is theorized serious end organ damage in SARS-CoV-2 infection is due to mast cell activation (13, 35, 36).

With respect to pregnancy, the primary skin disease to differentiate other than drug or infection is PEP (29, 37). PEP tends to occur late in the 3rd trimester or in the post-partum period usually in primigradas and begins within the abdominal striae. Unlike PEP, SARS-CoV-2 associated urticaria tends to resolve around 7 days (17). Newer reports have shown that chronic urticaria can develop particularly in young women after SARS-CoV-2 infection or SARS-CoV-2 vaccines (38, 39). The most common etiologic factors in the differential of urticaria in neonates and infants include infection, food, medications and atopy (40).

SARS-CoV-2 should be considered in the differential diagnosis of urticaria in pregnant women, neonates, and infants without constitutional symptoms due to the possibility of moderate to severe disease complications.

### Vesicular

**Figure 3:**
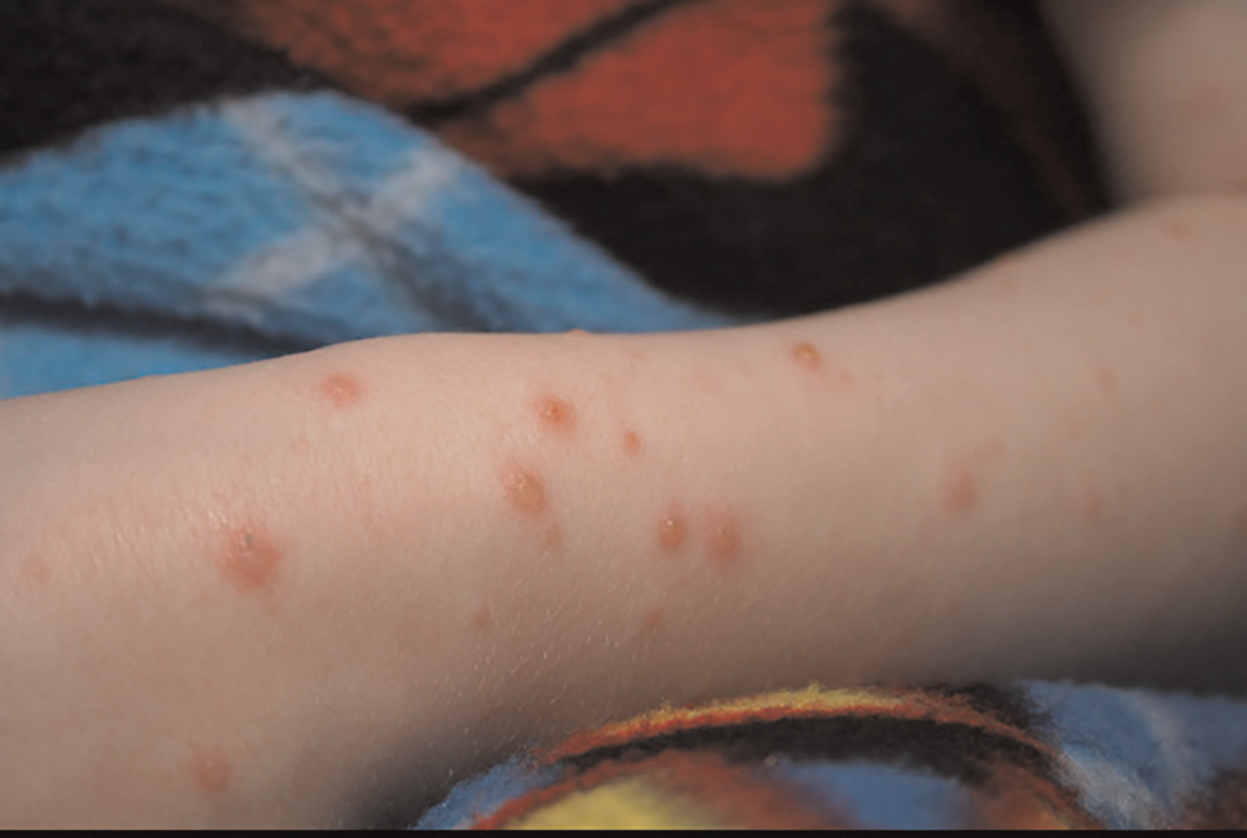
Vesicular pattern.

Vesicular eruptions in SARS-CoV-2 were first described as “varicella-like” in April 2020 by Marzano (20). Usually appearing on the trunk, scattered fluid filled blisters may appear localized or diffusely with or without purpura (Figure 3).

The prevalence ranges from 3.7%–15% occurring primarily in adults but has been reported in children (17, 19, 32, 41). SARS-CoV-2 vesicular exanthems are associated with moderate severity of illness and occur when patients are symptomatic in mid course of disease. Cases of early or late onset of vesicular lesions have been reported. The median duration of the eruption is approximately 8–10 days (17, 19, 20, 32). Vesicular eruptions associated with SARS CoV-2 are considered to be specific to the virus and may be useful diagnostically. In a systematic review, Jamshidi reports that vesicular lesions may be associated with neurologic symptoms including headache, dysgeusia, and confusion (42). The pathogenesis of vesicular lesions in SARS-CoV-2 is felt to be related to either a direct cytotoxic effect on dermal vessel endothelium or exaggerated release of cytokines (19, 41).

SARS-CoV-2 infection must be considered in the differential diagnosis of vesicular eruptions in pregnant women, neonates and infants. Causes to be eliminated include viral and bacterial infections, infestations, miliaria, irritant or contact dermatitis, and autoimmune diseases (19, 43, 44) (Table 1). Viruses such as herpes simplex virus (HSV), varicella zoster virus (VZV), measles, and rubella are associated with serious fetal sequelae and must be differentiated (45). HSV and VZV reactivation has been associated with SARS-CoV-2 infection with some patients developing more severe illness (46–48). Flaring of atopic dermatitis can present as vesicular lesions in pregnancy and infancy with secondary staphylococcal or HSV infection requiring immediate therapeutic intervention. Elevated IL-4 levels in pregnancy may be a factor in the exacerbation of atopic dermatitis (49). Common transient conditions in neonates may present with vesicular lesions (ETN, acropustulosis of infancy) or rare conditions such as Hyper IgE syndrome, or histiocytosis (50, 51). Pemphigoid Gestationis, a rare immunobullous disease in pregnancy, usually occurring in the 3rd trimester with periumbilical bullae should be included in the differential diagnosis of SARS-CoV-2 vesicular exanthems (29).

### Chilblain-like

**Figure 4:**
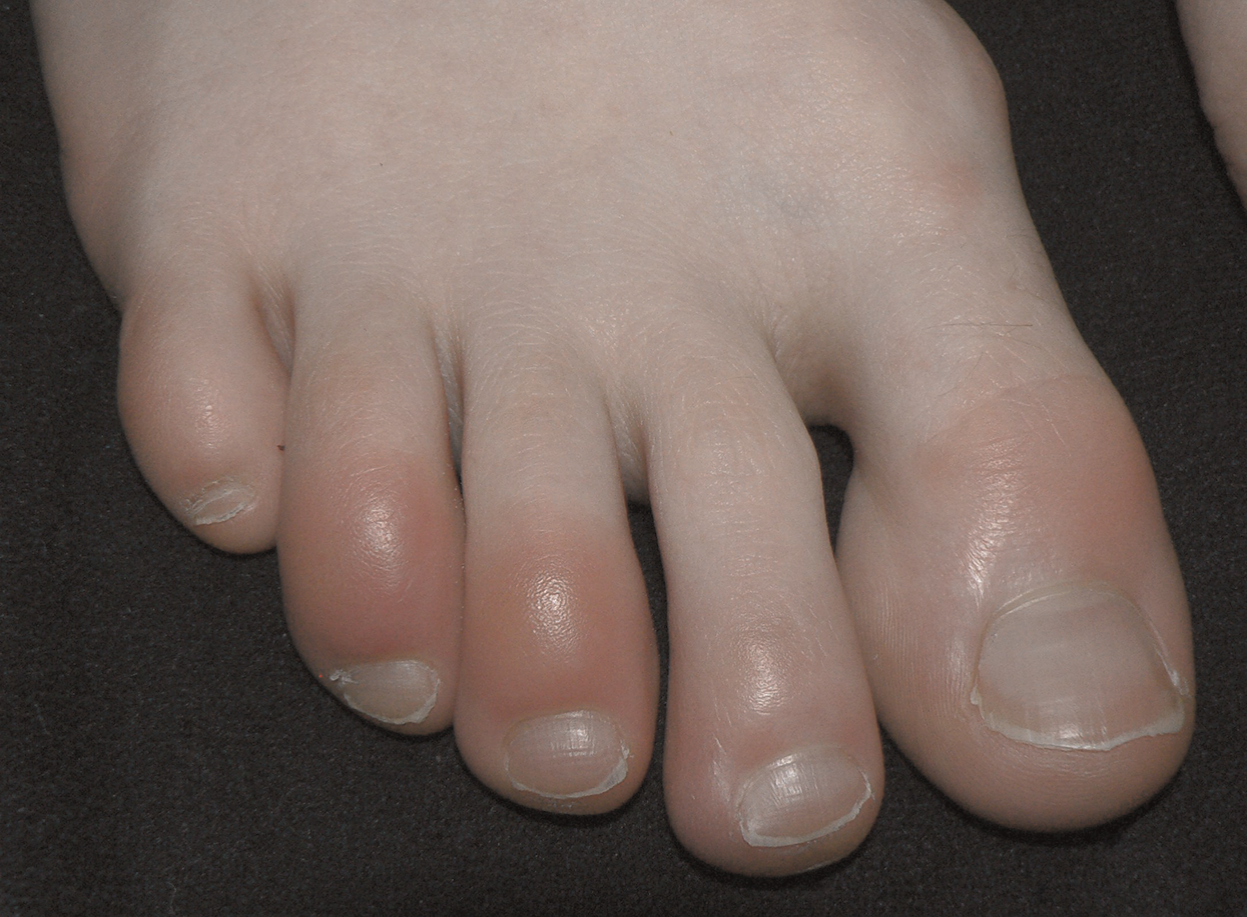
Chilblains pattern.

Chilblain-like or “pernio-like” vascular skin lesions of the hands and feet are probably the most recognized of all skin lesions associated with SARS-CoV-2. Multiple reports of non-blanching erythematous or violaceous lesions of the toes associated with SARS-CoV-2 infection help to coin the term “Covid toes” (19, 52) (Figure 4).

Usually seen in asymptomatic or mildly infected young adults and children, SARS-CoV-2 chilblain-like lesions tend to appear late in the course of infection. Numerous studies report the prevalence varies from 14.3%–72% (19). Chilblain and pernio diseases are vascular inflammatory reactive skin disorders to environmental stimuli such as cold exposure or damp humid environments. The hands and feet are primarily affected with a vasoconstrictive response and resultant erythematous, violaceous macules, papules or nodules of the fingers or toes. The most common symptoms are pain and pruritus. SARS-COV-2 associated chilblain-like lesions tend to last 1–2 weeks after the onset of symptoms and resolve without incident (32). Primary chilblain/pernio disease is idiopathic but secondary causes include autoimmune [systemic lupus erythematosus (SLE), antiphospholipid disease, Raynaud's disease] cryoglobulinemia, and hematologic diseases (52). There is controversy whether there is a direct association between SARS-CoV-2 infection and chilblain-like lesions. Colmenero (53) demonstrated the presence of SARS-CoV-2 in endothelial cells of pernio-like lesions by electron microscopy. On the contrary, many patients presenting with chilblain-like skin lesions tested negative with reverse transcription polymerase chain reaction test (RT-PCR), had negative serology, or were not tested at all (54, 55). This may be explained by robust protective levels of IFN-1 in younger patients or the significant variability of current testing. It has been proposed that chilblain-like lesions represent late manifestations of SARS-CoV-2 due to a delayed immunological reaction or an inappropriate type 1 interferon response (56). A literature review by Cappel et al. (56) suggested that the pathogenesis of SARS-CoV-2 chilblain-like lesions involves complex interactions between the virus, angiotension converting enzyme-2 (ACE-2), the renin-angiotension-aldosterone system, sex hormones, and interferon type 1 responses causing endothelial cell dysfunction (56–58).

Histopathology of skin lesions is similar to that found in idiopathic chilblains with epidermal necrotic keratinocytes, dermal edema, perivascular and perieccrine lymphocytic inflammation and microthrombi in the vasculature and endothelial cell inflammation (58).

Chilblain-like lesions may be the only sign of SARS CoV-2 in pregnant women, neonates and infants late in the disease course so it is important to properly diagnose this pattern and differentiate from other primary or secondary causes.

### Livedo

**Figure 5:**
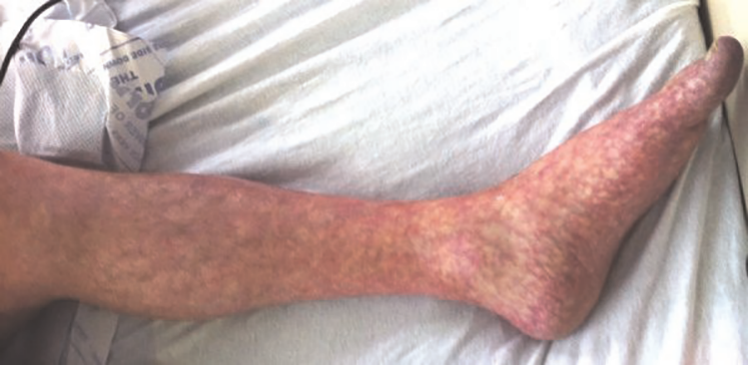
Livedo pattern.

Livedo patterns are less common manifestations of SARS CoV-2 ranging from 4%–6% (17, 19, 41). Infected patients presenting with livedo reticularis-like lesions tend to have milder disease and transiently clear over a period of 2 weeks with the average duration approximately 9–10 days (17, 19, 59, 60). The lesions appear as a mottled red-blue-purple net-like discoloration on the trunk, flexor forearm surface, dorsal hands and feet (Figure 5).

A pauci-inflammatory thrombogenic vasculopathy is noted on histopathology with serologic elevated D-Dimer levels (14, 61). It is theorized that the SARS CoV-2 virus directly infects endothelial or smooth muscle vessel cells causing low grade vascular inflammation and vasodilation. This process results in decreased blood flow with deoxygenated hemoglobin but no thromboembolism (60). Livedo reticularis must be differentiated from other causes including either physiologic, secondary, or idiopathic (Table 1).

Livedo racemosa is a more severe variant and is characterized by larger more widespread mottling of the skin that is generally secondary to a pathologic condition. Usually appearing in elderly patients with severe SARS-CoV-2 infection, the lesions can be transient or persistent and appear mid course during active symptoms. In contrast to livedo reticularis, patients presenting with livedo racemosa may develop severe coagulopathy and complications. Galvan Casas et al. (17) reported a mortality rate of 10% in patients presenting with livedo racemosa. Pathologically, vessels are partially occluded which leads to retiform purpura and complete vascular occlusion. Histologically, there is a micro thrombotic vasculopathy with possible dermal arterial thrombosis (62).The vasculopathy is thought to be due to direct viral effects or immune stimulation of the complement cascade with the release of proinflammatory cytokines (IL-6, IL-8, IFN γ, TNF-α) elevated D-Dimer levels, and fibrinogen degradation products which are associated with thrombosis and increased mortality (61, 62). Severity of SARS- CoV-2 illness in pregnant women may be correlated with elevated IFN γ, IL-6, and D Dimer levels (13).

In pregnant women, neonates and infants, livedo patterns can be seen associated with other hypercoagulable diseases including SLE and antiphospholipid antibody syndrome. Interestingly, antiphospholipid antibodies are found in SARS-CoV-2 patients with severe illness, livedo lesions, and severe thrombosis. In a study by Sangle et al. (63), widespread livedo reticularis is thought to be an independent factor of pregnancy complications in patients who have negative antiphospholipid antibodies with or without lupus. Rodriguez et al. (64) reported a case of an infant presenting with livedo racemosa and respiratory failure diagnosed as multisystem inflammatory syndrome (MIS-C). Given the high risk for severe complications of SARS-CoV-2, infected pregnant women, neonates and infants presenting with livedo patterns should closely be monitored and investigated for impending thrombotic events.

### Purpuric/vasculitic

**Figure 6:**
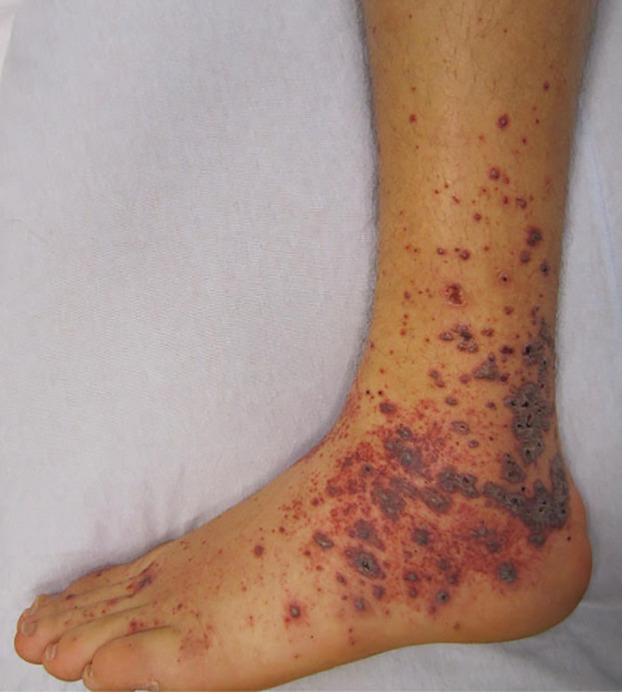
Purpuric/vasculitic pattern.

Vasculitic or purpuric lesions in SARS CoV-2 infected patients are associated with severe morbidity and mortality (17, 52). Less common than other patterns, various studies report a prevalence of 3%–8% and the lesions occurring during the symptomatic phase of infection(17, 19, 20, 32). The lesions appear as non-blanching hemorrhagic macules, patches, bullae, or palpable purpura on the extremities or acral areas (Figure 6).

Distal acral ischemia may occur leading to necrosis and gangrene of the digits (44). Diffuse spread of vasculitic lesions correlates with severe sequelae (65–67). Elevated D-Dimer and fibrinogen degradation products were found in patients with distal ischemia with some developing disseminated intravascular coagulation (68). Histologically, a true vasculitis is seen with a neutrophilic infiltrate within the small vessel walls, intense lymphocytic perivascular infiltrate, fibrin deposition, and endothelial swelling differentiating this pattern from livedo lesions (43).

Purpuric, petechial, or vasculitic lesions may be present in other viral infections in pregnant women, neonates and infants including hepatitis, human immunodeficiency virus, parvovirus B19 as well as bacterial infections, hematologic, autoimmune and nutritional disorders (45) (Table 1). Adverse drug reactions including antiviral agents are common causes of purpuric vasculitic eruptions in SARS Co-V-2 due to the multitude of therapeutics in severe illness (19). Systemic lupus erythematosus, thrombotic thrombocytopenic purpura, anti-neutrophil cytoplasmic antibody associated vasculitis, and parvo B-19 infections are included in the differential diagnosis of purpuric vasculitic lesions in pregnancy and can lead to serious perinatal and fetal complications (45, 69–71). Although rarely seen in this age group, Ig A vasculitis (Henoch Schonlein Purpura -HSP), Kawasaki's disease, and multisystem inflammatory syndrome should be considered in the differential diagnosis in neonates and infants with purpuric or vasculitic lesions and diagnosed promptly to prevent potential complications (72–74).

Pathogenesis of SARS-CoV2 petechiae/purpura/vasculitis is thought to be due to direct viral damage to endothelial cells causing endotheliitis and endothelial cell injury or a dysregulated inflammatory responses with immune complex deposition and massive cytokine release. Macrophage activation results and leads to the thrombotic lesions and events seen in SARS CoV02 coagulopathy (75).

Recognizing specific skin lesions may be prognostic in disease severity. Vasculitis, livedo racemosa, and distal ischemia are associated with more severe complications while chilblains-like lesions have the highest survival rates. The importance of identifying skin manifestations in SARS-CoV-2 infected pregnant women, neonates, and infants is imperative to allow early intervention and therapeutic management.

## Multisystem inflammatory syndrome

Early reports of SARS-CoV-2 infection indicated that children and neonates tended to be spared of severe associated complications. In 2020, a hyperinflammatory syndrome with characteristics similar to Kawasaki's Disease (KD) was reported in children with concurrent or post SARS-CoV-2 infection (73, 74). The syndrome was labeled as Multisystem Inflammatory Syndrome in Children (MIS-C) or Paediatric Inflammatory Multisystem Syndrome temporarily associated with SARS-CoV-2 (PIMS-TS) and in neonates as Multisystem Inflammatory Syndrome in Neonates (MIS-N) (76–78). Although the incidence is rare, MIS-C is a potentially life threatening variant leading to severe complications including cardiac injury, multiorgan failure and death, The most commonly involved organ systems are gastrointestinal, cardiovascular, hematologic, mucocutaneous, and respiratory. Overall, pediatric mortality due to MIS-C is reported at 1.9% but in neonates and young infants it may be as high as 9% (79).

MIS-C usually occurs in children aged 9 years (ranging 1 month to 20 years) and in neonates (MIS-N) from within 7 days to 27 days post birth (77–79). The CDC criteria for MIS-C/MIS-N includes persistent fever(not MIS-N), 2 organ system involvement, laboratory evidence of inflammatory markers, laboratory evidence of current or recent SARS-CoV-2 infection or maternal infection, and no other plausible disease causing the syndrome (79, 80).

Cutaneous and mucocutaneous lesions are present in approximately 73% of children with MIS-C (81). Maculopapular exanthems and conjunctivitis are the most commonly reported skin signs. Facial erythema or periorbital edema (“Heliotrope rash”), hand and foot edema, perineal erythema, desquamation, and cracked lips are noted features. Retiform purpura, targetoid lesions, urticaria and erythroderma have been described (81). Godfred-Cato et al. (82) reported skin rash was the most common presenting sign of MIS-C in infants less than 12 months of age and 32.9% of these infants required ICU admission. The appearance of a maculopapular rash in MIS-C may have prognostic implications depending upon the presentation. In a small study by Rekhtman (83), some MIS-C patients specifically presenting with maculopapular lesions had lower levels of inflammatory markers, less ICU admission, less mechanical ventilator support, and less serious consequences. In contrast, isolated purpuric and necrotic lesions have been noted in neonates with MIS-N with cardiogenic shock, elevated inflammatory markers, and multiorgan failure (84).

MIS-C patients with a Kawasaki's disease-like (KD-like) presentation have been reported. Similar lesions include conjunctival injection, hyperemic cracked lips, strawberry tongue, and coronary artery disease with severe complications. While classic KD patients tend to be younger (less than 5 years), MIS-C with KD-like disease patients are usually older (5–13 years) and present with more gastrointestinal symptoms. Both MIS-C/KD-like disease and KD patients may present with severe cardiac involvement but KD patients tend to have severe persistent sequelae. There are reports of persistent cardiac dysfunction in some MIS-C patients (85, 86).

The pathogenesis of MIS-C and MIS-N is unknown but theorized to result from autoantibody mediated complexes to SARS-CoV-2 infection through the respiratory or gut mucosa (87). Neonates may develop immune complexes derived from exposure to maternal antibodies (79). Others postulate that SARS-CoV-2 virus acts as a superantigen causing an exaggerated release of inflammatory mediators leading to cytokine storm (88). Consiglio (87) reported that MIS-C patients had lower levels of TNF-α and normal IL-6 levels both of which are elevated in acute SARS-CoV-2 infection casting doubt on the cytokine storm theory. The efficacy of intravenous immunoglobulin therapy in MIS-C supports an autoantibody mediated pathogenesis (87).

Although there are no diagnostic skin manifestations, cutaneous and mucosal lesions may be the presenting signs of MIS-C or MIS-N, early recognition of dermatological manifestations can lead to timely diagnosis and intervention (85).

## Less common skin manifestations of SARS-CoV-2

Unusual skin manifestations associated with SARS-CoV-2 have been reported with regard to maternal/fetal/infant health. Vertical transplacental transmission of SARS CoV-2 is rare but has been reported with possible associated skin manifestations. Generalized and local fetal skin edema diagnosed by ultrasound has been reported in pregnant women with SARS-CoV-2 infection (89). Associated elevated serological maternal levels of IL-6 and D-Dimer levels leading to cytokine stimulated inflammation or direct viral cytotoxicity is felt to alter the neonatal cutaneous microbiome resulting in fetal skin edema (89, 90). Necrotic lesions of the upper arm leading to amputation were noted in a neonate born to a SARS-CoV-2 infected mother and theorized that the virus may induce neonatal thrombotic events through exposure to maternal infection (91). Unusual orange discoloration of the skin was reported in a SARS-CoV-2 infected family in which yellow to brown macules were noted on the extremities of a newborn and yellow-brown discoloration of the palms and soles found on the other family members. The virus is thought to cause abnormalities in the conversion or transport of beta-carotene causing excess amounts to be deposited in the skin (92). Acute Hemorrhagic Edema of Infancy has been described in a SARS-CoV-2 infected infant which recurred 3 weeks after initial presentation and resolution (93). More evidence is needed to determine the relationship of neonatal infant skin eruptions and maternal SARS CoV-2 infections.

## Conclusion

There are limited reports on the relationship of SARS-Co-V-2 infection and related dermatologic manifestations in pregnant women, neonates and infants but reports have demonstrated this patient population is at high risk for SARS-Co-V-2 complications. Skin lesions may be the first sign of infection and be prognostic for disease severity. Severe morbidity and mortality have been associated with the appearance of purpuric and vasculitic lesions and less commonly with chilblains-like lesions. By identifying skin manifestations in SARS-Co-V-2 infected pregnant women, neonates, and infants, asymptomatic infections may be properly diagnosed, disease transmission prevented, and severe disease complications averted. It is important to differentiate other skin diseases which can flare during pregnancy or in the neonatal/infancy period due to physiologic immunological shifts. The collaboration between dermatology, obstetrics and gynecology, neonatology, pediatrics, and infectious disease can optimize perinatal/maternal-fetal-infant health care in the diagnosis and treatment of SARS- CoV-2.
