# Supplementary figures and images for: Perinatal/maternal-fetal-infant dermatologic manifestations of SARS-CoV-2. An Overview and Implications for diagnosis, treatment, and prognosis
Source: Front Pediatr. 2022 Dec 2;10:1071839. doi: 10.3389/fped.2022.1071839 (PMC9755859; doi:10.3389/fped.2022.1071839)

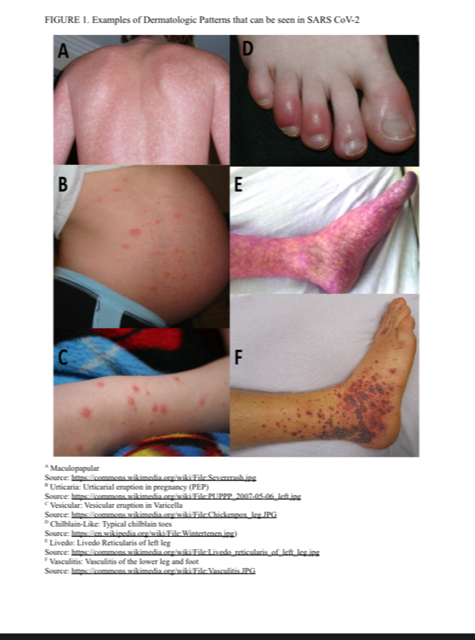

Supplement: Supplementary file 1 [file Image1.png]

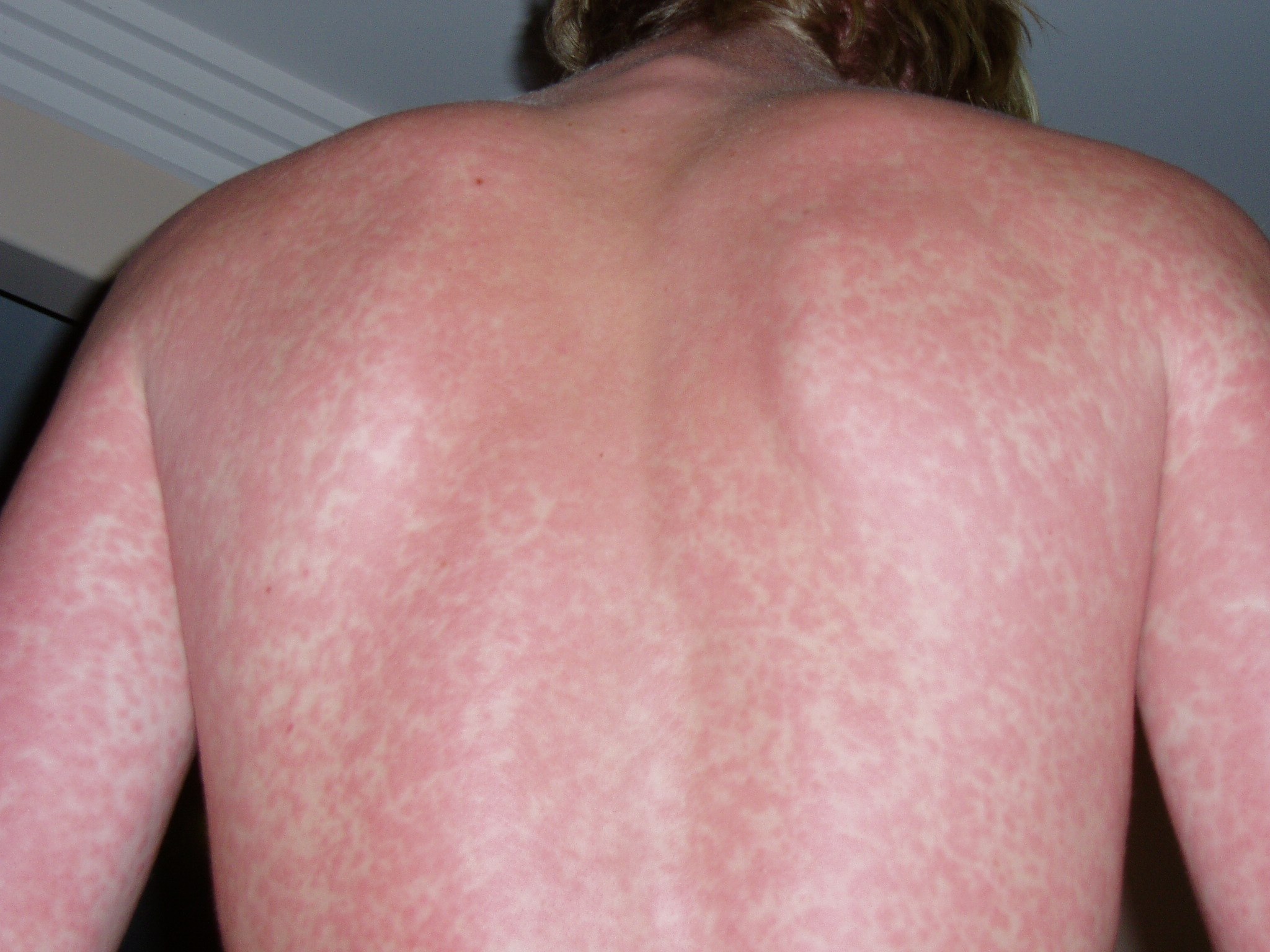

Supplement: Supplementary file 2 [file Image2.jpeg]

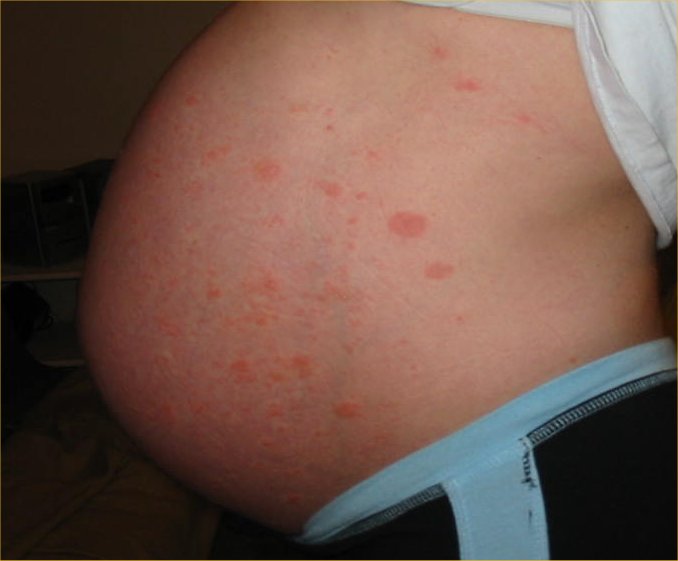

Supplement: Supplementary file 3 [file Image3.jpeg]

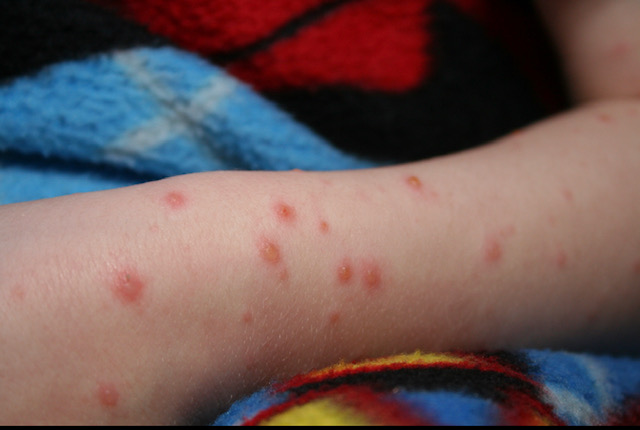

Supplement: Supplementary file 4 [file Image4.jpeg]

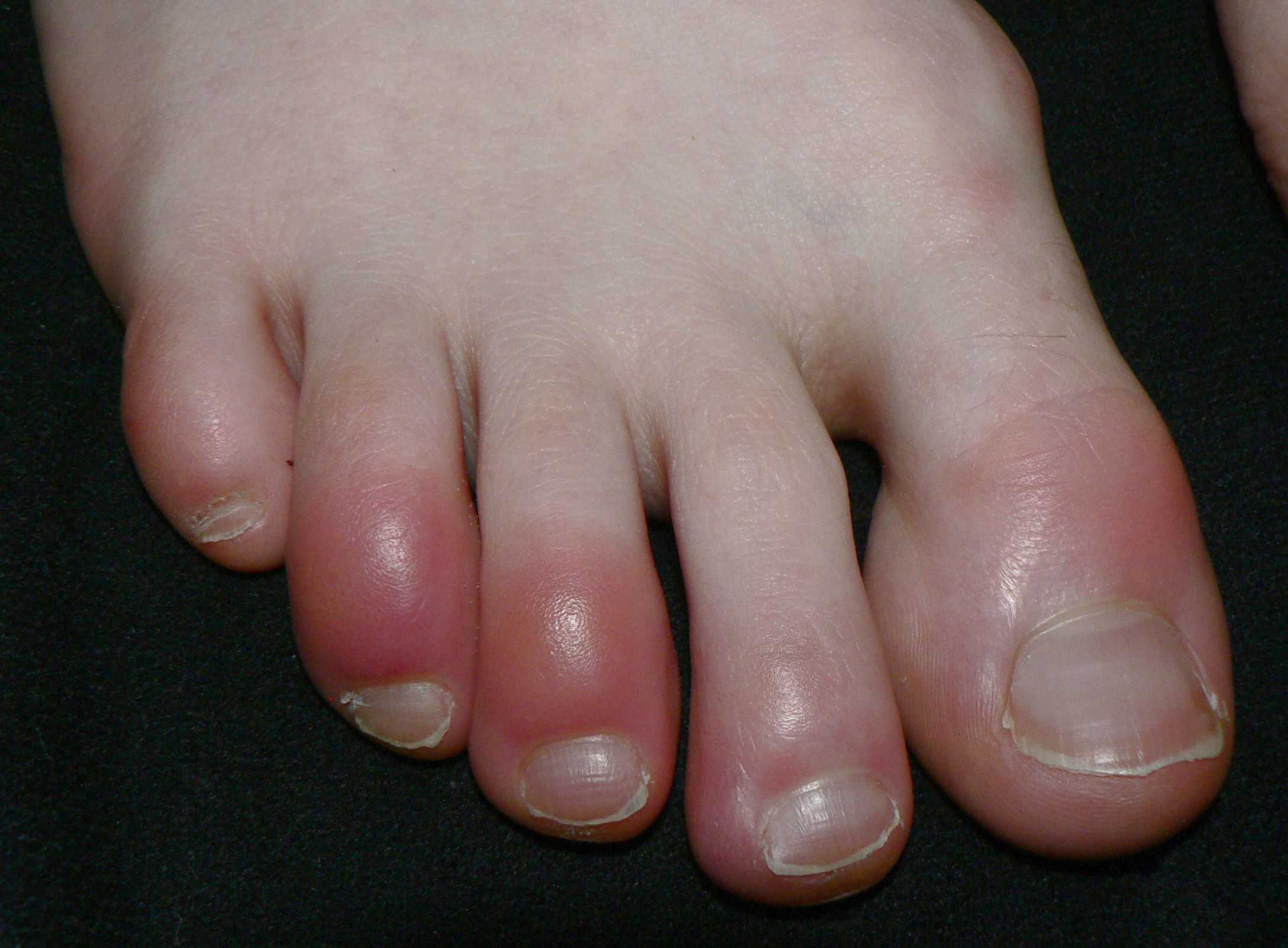

Supplement: Supplementary file 5 [file Image5.jpeg]

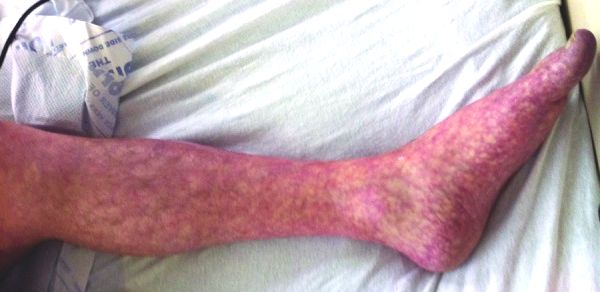

Supplement: Supplementary file 6 [file Image6.jpeg]

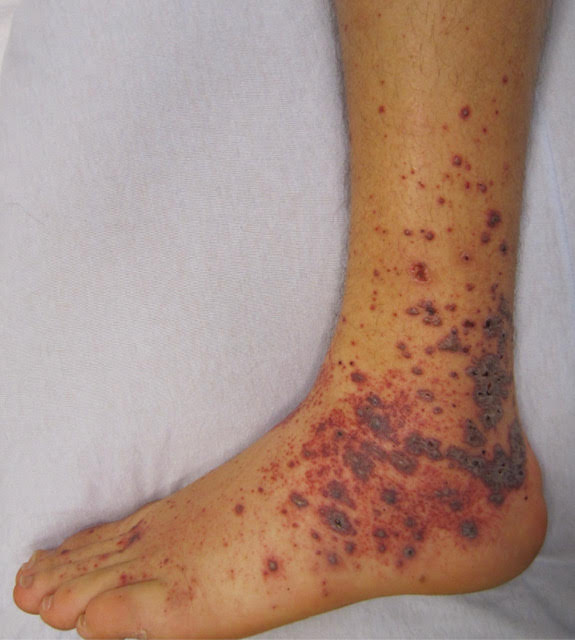

Supplement: Supplementary file 7 [file Image7.jpeg]
